# PUF-Immobilized *Bjerkandera adusta* DSM 3375 as a Tool for Bioremediation of Creosote Oil Contaminated Soil

**DOI:** 10.3390/ijms232012441

**Published:** 2022-10-18

**Authors:** Katarzyna Struszczyk-Świta, Piotr Drożdżyński, Karolina Murawska, Olga Marchut-Mikołajczyk

**Affiliations:** Institute of Molecular and Industrial Biotechnology, Faculty of Biotechnology and Food Sciences, Lodz University of Technology, Stefanowskiego 2/22, 90-537 Lodz, Poland

**Keywords:** creosote oil, soil bioremediation, white-rot fungi, *Bjerkandera adusta*

## Abstract

Creosote oil, a byproduct of coal distillation, is primarily composed of aromatic compounds that are difficult to degrade, such as polycyclic aromatic hydrocarbons, phenolic compounds, and N-, S-, and O-heterocyclic compounds. Despite its toxicity and carcinogenicity, it is still often used to impregnate wood, which has a particularly negative impact on the condition of the soil in plants that impregnate wooden materials. Therefore, a rapid, effective, and eco-friendly technique for eliminating the creosote in this soil must be developed. The research focused on obtaining a preparation of *Bjerkandera adusta* DSM 3375 mycelium immobilized in polyurethane foam (PUF). It contained mold cells in the amount of 1.10 ± 0.09 g (DW)/g of the carrier. The obtained enzyme preparation was used in the bioremediation of soil contaminated with creosote (2% *w*/*w*). The results showed that applying the PUF-immobilized mycelium of *B. adusta* DSM 3375 over 5, 10, and 15 weeks of bioremediation, respectively, removed 19, 30, and 35% of creosote from the soil. After 15 weeks, a 73, 79, and 72% level of degradation of fluoranthene, pyrene, and fluorene, respectively, had occurred. The immobilized cells have the potential for large-scale study, since they can degrade creosote oil in soil.

## 1. Introduction

Creosote oil is a complex mixture of organic compounds obtained from the high-temperature distillation of coal tar. It is composed of approximately 85% polycyclic aromatic hydrocarbons (PAHs), 10% phenolic compounds, and 5% heterocyclic nitrogen, sulfur, and oxygen compounds [1,2]. Creosote is widely used as a wood preservative, particularly to prevent rotting in railway sleepers, power line poles, ship hulls, and timber bridges. This is a result of its sporicidal, insecticidal, and antifungal activities [1,2,3]. In 2011, the European Commission for the first time approved the use of this oil as a biocide for wood preservation [4]. However, creosote oil is considered a carcinogen, and some of the compounds in the oil are found to be persistent, bioaccumulative, and highly toxic [1,2,5]. Since there are no alternatives, this substance is still used despite its negative effects on the environment and human health. Existing factories that impregnate wood materials with creosote create heavily contaminated areas, both in terms of water and soil. The most common PAHs in creosote or oil-hydrocarbons-contaminated soil are acenaphthene, anthracene, benz[a]anthracene, fluoranthene, fluorene, 2-methylnaphthalene, naphthalene, phenanthrene, and pyrene [6], but also chrysene and benzo(a)pyrene [7]. Because such pollution must be removed from the environment as soon as possible, efficient bioremediation solutions for areas polluted with creosote oil are being investigated. Bioremediation involves the use of microorganisms which, as part of their metabolism, carry out various biochemical processes to remove pollutants from the environment. Autochthonous soil microorganisms are crucial in the self-cleansing processes. However it may be necessary to use a bioaugmentation technique for persistent, cancerogenic, and toxic pollutants like creosote oil. This method is based on the introduction of a specially designed consortia of microorganisms, or single microorganisms, which exhibit the ability to degrade a particular pollution into a contaminated environment [8]. Bioaugmentation allows for a faster and more effective biodegradation of complex organic compounds into less toxic or completely safe compounds [9,10,11]. Unfortunately, in the case of PAHs, microorganisms rarely carry out the complete mineralization process, and the purifying procedure itself takes time. Therefore, techniques to enhance bioremediation are still needed. Fungal bioremediation may be a solution to this problem, since some fungi have the capacity to degrade recalcitrant organic pollutants, such as PAHs [12,13].

Although the degradation of creosote oil is difficult, it has been proven that some microorganisms can carry out its degradation in soil. These are mainly bacteria from the genera *Pseudomonas*, *Serratia*, and *Sphingomonas* [2]. The soil purification process can be also supported by biopreparation containing *Yarrowia lipolytica* yeast [14]. In recent years, there has been a rising interest in the use of fungi for biodegradation. This is due to the fact that these microbes may degrade mixtures of chemical compounds at the same time. Special attention is given to the white rot fungi (WRF) [15]. These microorganisms are able to produce ligninolytic enzymes, of which laccase (Lac, EC 1.10.3.2), manganese-dependent peroxidase (MnP, EC 1.11.1.13), and lignin peroxidase are the most important (LiP, EC 1.11.1.14). Because of their low substrate specificity, these enzymes catalyze not only the oxidation of lignin, but also the oxidation of a wide range of structurally varied chemicals that are difficult to degrade, such as PAHs [16,17,18,19], synthetic dyes [20,21], chlorinated organic compounds [22], pesticides [23,24], or residues of drugs [25]. Because of their low substrate specificity, these proteins are also beneficial in the decomposition of creosote. The following WRFs were used in the process of this oil biodegradation: *Peniophora incarnata* [26], *Pleurotus* [27], *Pleurotus ostreatus* [28,29,30], *Irpex lacteus* [30,31], *Phanerochaete chrysosporium*, and *Bjerkandera adusta* [31]. The WRF’s pathogenicity to plants and other organisms is the main obstacle to its application in the bioremediation of creosote oil-contaminated soil. Therefore, only WRF species that are not harmful to living organisms should be selected.

The objective of the study was to combine the immobilization approach with fungal biodegradation to develop an efficient way of cleaning creosote-contaminated soil on a laboratory scale. The bioremediation of creosote-contaminated soil was carried out using the mycelium of *Bjerkandera adusta* DSM 3375 immobilized in polyurethane foam (PUF).

## 2. Results

### 2.1. Immobilization of the Mycelium of B. adusta DSM 3375 in a Polyurethane Carrier

**Figure 1:**
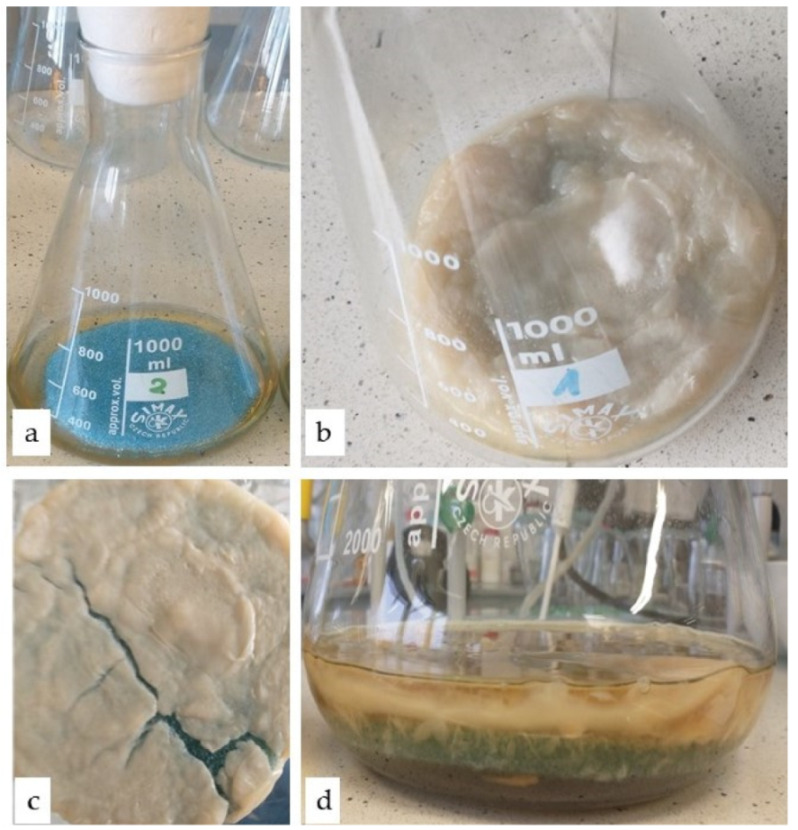
Immobilization of *B. adusta* DSM 3375 mycelium in PUF: (**a**) FiltrenTM30 foam placed in the medium, photo taken before starting the cultivation; effect of immobilization of mycelium in foam: (**b**,**c**) shake culture, (**d**) stationary culture.

Because of their high porosity, chemical inertness, physical stability, and flexibility, polyurethane foams (PUFs) are an excellent matrix for the immobilization of microbial cells, particularly filamentous fungi and yeast. The immobilization of *B. adusta* DSM 3375 was accomplished by inserting the PUF carrier into the culture medium (Figure 1a) and growing the mold, during which the mycelium overgrew the carrier’s pores. It was found that in the shaken culture, the mycelium grows both inside and on the surface of the foams (Figure 1b,c). In stationary culture, the mycelium only grows on the carrier’s surface, forming a thick layer that can be easily peeled off the foam (Figure 1d).

**Figure 2:**
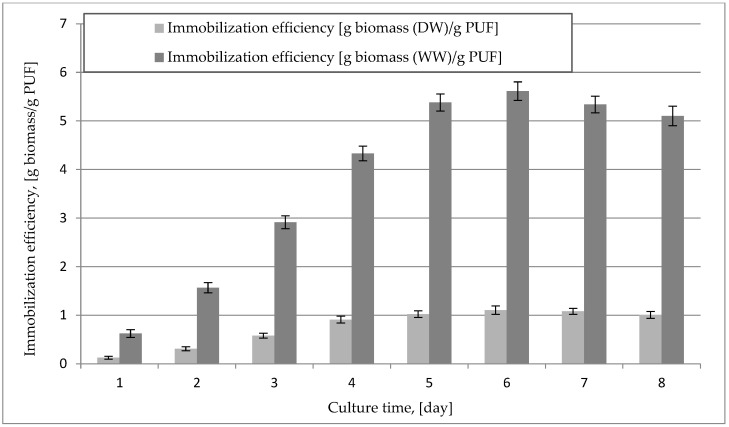
The efficiency of immobilization of *B. adusta* DSM 3375 mycelium in a polyurethane carrier during 8-day cultivation.

The immobilization of the mycelium of *B. adusta* DSM 3375 in PUF was most effective on the sixth day of cultivation. During this time, the best mycelial overgrowth of the foams was found, and the highest mass of mycelium was obtained in 1 g of foam, i.e., 1.1 g of dry matter. As a result of cell autolysis, the amount of biomass in the carrier decreased with time. Figure 2 illustrates the correlation of immobilized mycelium in PUF at the time of mold cultivation.

**Figure 3:**
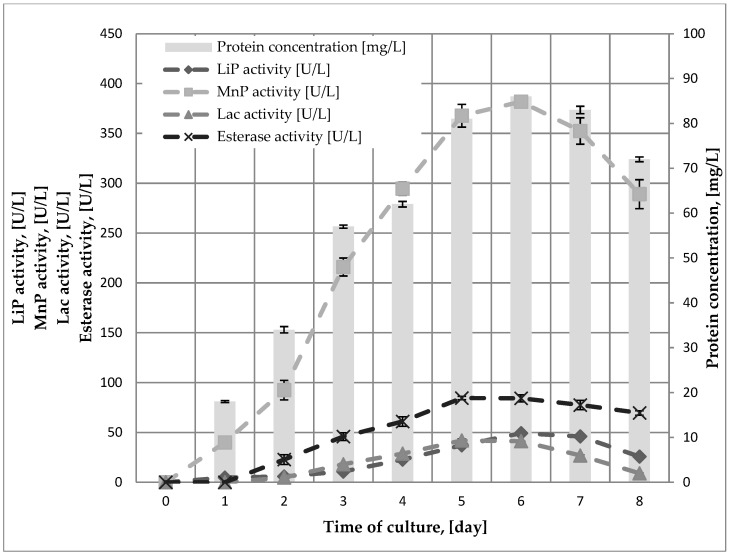
The activity of extracellular enzymes and the protein concentration of *B. adusta* DSM 3375 during 8-day cultivation.

During growth, the *B. adusta* DSM 3375 strain was found to produce extracellular oxidoreductases such as Lac, LiP, and MnP, as well as esterases (EC 3.1.1.x) from the hydrolase class. Figure 3 shows the correlation between the activity of these enzymes and the time of the *B. adusta* DSM 3375 culture.

After the sixth day of culture, the highest concentration of extracellular protein was determined (83 mg L^−1^). The maximum value of Lac and esterase activity occurs on the fifth day of cultivation and is 42 U L^−1^ and 84.5 U L^−1^, respectively. The fungus also produces MnP and LiP, the activity of which peaked on the sixth day of cultivation at 382 U L^−1^ and 49 U L^−1^, respectively. As a result, it was confirmed that the immobilized mycelium of the experimented strain was effective in the production of such enzymes.

After confirming the effectiveness of *B. adusta* DSM 3375 cell immobilization in PUF and their ability to produce extracellular ligninolytic enzymes and esterases in a bound form, the obtained preparations were used in the bioremediation of creosote-contaminated soil.

### 2.2. Creosote Removal Using PUF-Immobilized B. adusta DSM 3375 Cells

Table 1 shows the results of removing creosote oil from soil during bioremediation using *B. adusta* DSM 3375 mycelium immobilized in PUF carriers. After 5, 10, and 15 weeks of bioremediation, mycelium immobilized in the Filtren TM30 carrier resulted in creosote losses of 19, 30, and 35%, respectively (taking into account the control samples). The percentage loss of aromatic hydrocarbons was calculated using the UV light absorption properties of this class of compounds. The spectrum was measured in the wavelength ranges = 297, 299, and 302 nm, which correspond to the UV absorption maxima of PAH examples: pyrene, fluorene, and fluoranthene, respectively, present in the pure hydrocarbon mixture used for soil contamination. The results are shown in Table 2.

The results showed that after 35 days of bioremediation, the levels of fluoranthene, fluorene, and pyrene in the soil were reduced by 66, 58, and 58%, respectively. For fluoranthene, fluorene, and pyrene, respectively, the decomposition of PAHs in the soil by *B. adusta* DSM 3375 enzymes is slower and reaches levels of 79, 72, and 73 percent after 105 days. The losses of PAHs at the end of the cleaning process in control samples containing PUF without mycelium were 8, 7, and 9 percent, respectively. Creosote oil may also be adsorbed in the PUF. The third phase of the investigation thus focused on the ability of PUF or mycelium to absorb creosote. The loss of each of the measured aromatic hydrocarbons in the control samples without PUFs did not exceed 7.5–8.5%. It was confirmed that the amounts of substances extracted from the preparation were negligible and had no effect on the effectiveness of bioremediation.

## 3. Discussion

To date, small cube-shaped PUF samples have been used to immobilize *B. adusta* cells for lignin-degrading enzyme production [32,33,34,35]. However, using the enzyme preparation in this form in the field trials of bioremediation for creosote-contaminated soil would make its removal difficult after the clean-up process is completed. As a result, in the research that was reported, attempts were made to immobilize the mycelium in bigger PUFs. These are preliminary studies that will lead to the development of a method for producing a polyurethane mat filled with *B. adusta* cells that can be easily removed when bioremediation is completed.

Based on the results, it was demonstrated that the *B. adusta* DSM 3375 effectively overgrows the used carrier during shaking cultivation in a medium containing, in addition to nutrients, polyurethane foam. MnP was the most active of the extracellular enzymes produced by the strain (382 U L^−1^). It was similar to Tripathi et al.’s [36] work, in which non-immobilized *B. adusta* cells produced MnP, LiP, and Lac proteins, with the former exhibiting the highest activity (215 U L^−1^; the culture was performed in a medium rich in nutrients under stationary conditions). The activities of the other two enzymes, LiP, and Lac, were 17 and 5.5 U L^−1^, respectively. Importantly, *B. adusta* DSM 3375 generates esterases in addition to ligninolytic enzymes while growing in PUF. All of these enzymes are essential for the degradation of hydrocarbons, particularly aromatic hydrocarbons [8,37,38]. It can be concluded that the obtained biopreparation will be effective in the degradation of creosote oil present in contaminated soil.

As previously stated, *B. adusta* cells were effectively immobilized in porous, cube-shaped PUF carriers. It has been confirmed that cells immobilized in this manner produce extracellular enzymes such as Lac, MnP, and LiP, which are capable of degrading and discoloring lignin, sparking interest in their large-scale usage in lignin depolymerization and toxic pollutant degradation [32,33]. The same researchers noted that *B. adusta* IFO 4983 mycelium immobilized in PUF produces significantly more ligninolytic enzymes than unbound mycelium [32]. The activities of the abovementioned enzymes were 300, 250, and 50 U mL^−1^ for LiP, MnP, and Lac, respectively, whereas for enzymes produced by unbound mold, they were only 22, 8, and 1.8 U mL^−1^. Significant differences in the reported activity values for the *B. adusta* DSM 3375 and IFO 4983 strains are most likely the result of a different assay procedure. Another application of immobilized *B. adusta* mycelium in PUF is the decolorization of wastewater and of two solutions containing synthetic recalcitrant compounds made with tannic and humic acid. After one week of treatment, it was confirmed that the preparation of *B. adusta* MUT 2295 removed the color of the effluents by 49, 25, and 42 percent in crude effluent, tannic acid, and humic acid, respectively. This fungus is capable of degrading the aforementioned pollution. The strain is metabolically active during the decolorization process, as evidenced by the production of MnP [34].

The main goal of this study was to assess the potential of PUF-immobilized *B. adusta* DSM 3375 cells for the biodegradation of creosote hydrocarbons in soil. The efficiency of bioremediation in the tested soil was evaluated by analyzing three different selected examples of PAHs—fluoranthene, fluorene, and pyrene. Based on the loss of these compounds, the ability of the strain to biodegrade creosote was determined. After 35 days of bioremediation, the concentration of fluoranthene, fluorene, and pyrene in the soil decreased by 66, 58, and 58 percent, respectively. The results account for the abiotic loss of PAHs, along with those caused by the activity of autochthonous soil microorganisms. However, because no control tests under sterile conditions were conducted, it is not possible to provide data for hydrocarbons loss caused only by abiotic factors. Following our prior investigations [39], we conducted the bioremediation of creosote-contaminated soil in conditions simulating the natural environment.

Extracting and analyzing the PAHs present in soil is difficult due to the structures of these compounds, their hydrophobicity, and the ability to accumulate on the surface of solid materials. Acenaphthene, anthracene, benz[a]anthracene, fluoranthene, fluorene, 2-methylnaphthalene, naphthalene, phenanthrene, pyrene [6], but also chrysene and benzo(a)pyrene [7], are the most common PAHs in creosote or oil-hydrocarbons polluted soil. However, the distribution of the creosote phase and the subsequent spread of this pollutant in the environment should be considered when determining PAHs in environmental samples. While investigating the occurrence of PAHs in creosote-contaminated soil, Cavalcanti et al. [40] found just three hydrocarbons: napthalene, phenanthrene, and pyrene.

The degree of PAHs degradation in soil is determined by a variety of factors, including soil physicochemical properties, contamination type, its concentration and aging, the presence of degrading microorganisms, and also PAH bioavailability [41]. In contrast to bacteria, fungi decompose polyaromatic hydrocarbons through a different mechanism. The majority of fungi cannot use PAHs as a source of carbon and energy; however, they may co-metabolize these compounds to a range of oxidized products, sometimes including CO_2_ [42,43]. In case of the WRF, their extracellularly secreted ligninolytic enzymes are able to diffuse toward the immobile PAHs, making them useful in the initial attack on these contaminants in soil. The transformation of polyaromatic hydrocarbons by ligninolytic enzymes occurs via the production of hydroxyl free radicals through the donation of one electron, which oxidizes the PAH ring. As a consequence, acids and PAH-quinones are produced in place of dihydrodiols [44]. The mineralization of PAHs by ligninolytic fungi involves a combination of ligninolytic enzymes, cytochrome P450 monooxygenases, and epoxide hydrolases [43].

As previously stated, PAHs have high hydrophobicity, making them less available for biological uptake. This problem can be overcome by the use of bio/surfactants, the actions of which reduce the interfacial surface tension and thus increase the solubility of aromatic hydrocarbons [2,45,46]. Microorganisms are also able to produce biosurfactants during PAH degradation, making them more accessible [47]. As a result, aside from the enzymes *B. adusta* DSM 3375, other factors might have affected hydrocarbon loss in the soil.

The ability of *B. adusta* immobilized in PUF to bioremediate creosote-contaminated soil has not yet been studied. However, Byss et al. [48] achieved a relatively good bioremediation efficacy of creosote oil-contaminated soil for WRF, such as *Pleurotus ostreatus* and *Irpex lacteus*. According to the test results, even though the molds were not immobilized, *P. ostreatus* and *I. lacteus* biodegraded all PAHs at rates of 55–67% and 27–36%, respectively, after 120 days. The ability of *B. adusta* BOS55 to degrade PAH compounds was also confirmed by Valentn et al. [16], who used this strain for the bioremediation of PAH-contaminated soil (dibenzothiophene, fluoranthene, pyrene, and chrysene). After a 30-day process, the degradation rate of pyrene was 43 percent, chrysene, 43 percent, fluoranthene, 41 percent, and dibenzothiophene, 65 percent. Similar experiments were carried out by Andriani and Tachibana [37]. In these studies, *B. adusta* SM46 mycelium immobilized on rice straw was used in the bioremediation of PAH-contaminated sand. After 30 days, the degradation rates for naphthalene, phenanthrene, chrysene, and benzo(a)pyrene were 94%, 70%, 55%, and 63%, respectively. The strain immobilized on this carrier produced the Lac, MnP, and LiP enzymes.

## 4. Materials and Methods

### 4.1. Microorganism

The filamentous fungus strain of *Bjerkandera adusta* DSM 3375 was obtained from the Leibniz Institute DSMZ-German Collection of Microorganism and Cell Cultures GmbH (Braunschweig, Germany). The strain was stored at 4 °C on a solid medium slant containing malt extract 3% (*w/v*), soy peptone 0.3% (*w/v*), and agar 1.5% (*w/v*). All components of the culture medium were obtained from BTL Ltd. (Lodz, Poland).

### 4.2. Materials

Grade C creosote oil was supplied by DAW-BYTOM (Bytom, Poland). The soil sample was collected from the vicinity of the A1 motorway (51°46.6″ N; 19°37′58.3″ E), central Poland. The basic physical and chemical properties of fresh soil samples were determined according to the PN-R-04032:1998 [49], PN-ISO 11465:1999 [50], PN-EN 15309:2010 [51], PN-R-04028:1997 [52], and PN-EN ISO 10390:2022-09 [53] standards, and presented in Table 3. The soil was not sterilized, to ensure the most similar conditions to the environmental ones.

### 4.3. Immobilization of B. adusta DSM 3375 Mycelium in a Polyurethane Carrier

The *B. adusta* DSM 3375 strain was cultivated for 6 days at 30 °C, with agitation at 80 rpm. The liquid culture medium consisted of malt extract 3% (*w*/*v*) and soy peptone 0.3% (*w*/*v*), pH 5.6. The components of the culture medium were obtained from BTL Ltd. Poland. The cultivation was performed in 1 L Erlenmeyer flasks with 20% (*v*/*v*) of the flask filled with the medium. Before sterilization, open cell reticulated polyurethane foam (circle 15 cm in diameter and 1 cm high) was added to the culture medium (Filtren TM30, based on polyether polyol, pore diameter 1.6–2.2 mm, density 19–22 kg/m^3^, final extension 100%, tensile strength 80 kPa, purchased from Recticel, Brussels, Belgium). The inoculum was prepared by washing spores of *B. adusta* DSM 3375 from agar slants with 4 mL sterile saline and adding this to the sterile medium at 1% (*v*/*v*). After cultivation, the PUF-bound mycelium was thoroughly washed 3 times with distilled water. Subsequently, the water from the mycelium was removed on a mechanical press, then immobilized mycelium was used for the bioremediation of soil contaminated with creosote oil. The samples of the preparation were also dried to a constant mass at a temperature of 105 °C, to determine the amount [g] of mycelium bound with the [g] PUF.

On the other hand, the extracellular protein concentration and activity of enzymes were determined in the culture medium after its centrifugation (10,000 rpm, temperature 4 °C). The activities of the following enzymes were assayed: Lac, MnP, LiP, and carboxylic ester hydrolases.

Protein concentration was determined according to the protocol of Bradford [54], and expressed as the amount of protein, mg L^−1^. The enzymatic activity of Lac was determined using the method described by Han et al. [55], using the oxidation of 2,2′-azinbis-(3-ethylbenzothiazoline-6-sulfonate) (ABTS, e420, 36,000 M^−1^ cm^−1^) to a more stable and favorable form of the cationic radical. One activity unit (U) was defined as the amount of enzyme that oxidized 1 μmol of ABTS per minute at 25 °C. Determination of the MnP activity was based on the oxidation of Mn^2+^ to Mn^3+^ ions in the presence of hydrogen peroxide. The oxidized product forms a colored complex with sodium potassium tartrate (e238, 6500 M^−1^ cm^−1^), [56]. One unit (U) of enzyme activity was defined as the amount of required enzyme to produce 1 µmol product per minute under the experimental conditions. The LiP activity was measured via the oxidation of veratryl alcohol (VA) to veratryl aldehyde (e310, 9300 M^−1^ cm^−1^), [57]. One unit (U) of LiP was defined as the amount of enzyme necessary to oxidize 1 μmol VA per minute at 25 °C. The esterolytic activity was determined according to Szczęsna-Antczak [58], assayed via the hydrolysis of p-nitrophenol acetate (p-NPA, e399, 1.32 × 10^−4^ M^−1^ cm^−1^) at pH 7, and expressed in standard units (U = µmol p-NPA min^−1^).

### 4.4. Bioremediation of Soil Contaminated with Creosote Oil

The bioremediation of soil contaminated with creosote oil (2% *v*/*w*) was carried out at a temperature of 24 °C for 15 weeks. The soil moisture was kept at 20% (*w*/*w*), taking into account the soil’s water holding capacity. The pH of the soil was not adjusted. To perform the bioremediation of soil contaminated with creosote oil, the moist mycelium of *B. adusta* DSM 3375 immobilized in a polyurethane carrier (approximately 20 g (WW) with an average moisture content of 80.15 ± 0.26%) was introduced per kg of soil. The study included two types of control samples, which were non-sterile soil samples containing the same amount of creosote oil (2% *v*/*w*). Empty PUF (without mycelium) was introduced in the first control, while the second control contained only soil with creosote (without PUF-immobilized *B. adusta* DSM 3375 mycelium or empty PUF). The remaining conditions of bioremediation in the control samples were identical to those in the proper samples. The efficiency of bioremediation was determined by measuring the loss of creosote in soil samples every 5 weeks (extraction using the Soxhlet method).

### 4.5. UV-VIS Spectroscopy

The loss of aromatic hydrocarbons was determined in the pollutant obtained after extraction with dichloromethane in the Soxhlet apparatus. Then, the samples were diluted 28,000× in the dichloromethane. The loss of aromatic hydrocarbons was examined for the examples of fluoranthene, fluorene, and pyrene, with absorption maxima at the wavelengths of λ = 302, 299, 297 nm, respectively, on the T80 + UV/VIS spectrophotometer by PG Instruments Ltd (Leicestershire, UK). The results were analyzed and expressed as a percentage loss of aromatic hydrocarbons using a pure hydrocarbon mixture as a control.

### 4.6. Statistical Analysis

The experiments were performed in triplicate. Statistical error were calculated using Microsoft Excel 2013 software. The calculation of mean values and standard deviations, and the analysis of variance (single factor ANOVA) were conducted using Statistica 10.0 software. The differences between individual means and the control mean were tested using Tukey’s test. Significance was set at *p* = 0.05.

## 5. Conclusions

The fundamental problem with using creosote oil are the toxicity, carcinogenicity, and persistence of its main constituents, PAHs. Creosote is a difficult-to-degrade substance, due to its low solubility in water and high affinity for soil particles. So far, no efficient technique for removing it from contaminated areas has been developed. The goal of the study was to use immobilized *Bjerkandera adusta* DSM 3375 mycelium to develop a technique for the breakdown of creosote oil in soil. In an immobilized form, this mold produces active oxidoreductive enzymes (laccase, lignin peroxidase, and manganese-dependent peroxidase) and esterases capable of PAHs decomposition. Because the amount of introduced creosote, including PAHs, was significantly reduced, it may be concluded that *B. adusta* DSM 3375 produced the same enzymes during soil bioremediation in situ. A method for immobilizing mold cells in polyurethane foam with dimensions that allow for its easy removal from the soil after the bioremediation process was developed. The research carried out is the basis for the development of mycelium immobilization technology in mats composed of porous polyurethane carriers and their application in field trials in plants using creosote oil for wood impregnation. Because no ecological alternative for creosote has yet been developed, soil pollution with this substance will still occur. The proposed bioremediation method is a very promising approach for the clean up of PAH-contaminated environments.

## Figures and Tables

**Table 1:** Weight loss of creosote oil during 15-week bioremediation of soil by PUF-immobilized mycelium of *B. adusta* DSM 3375.

Bioremediation Time [Week]	Mass of PUF-Immobilized Mycelium [g DW/kg of Soil]	Mass of Mycelium [g DW/kg of Soil]	Loss of Creosote Oil [%] *
5	3.898 ± 0.101	1.997 ± 0.088	26.10 ± 1.50
Control sample—PUF without mycelium (1.911 ± 0.03 g/kg of soil)			6.70 ± 0.50
10	3.698 ± 0.104	1.922 ± 0.042	39.03 ± 0.98
Control sample—PUF without mycelium (1.784 ± 0.07 g/kg of soil)			8.78 ± 0.62
15	3.944 ± 0.080	2.036 ± 0.051	47.88 ± 1.27
Control sample—PUF without mycelium (1.917 ± 0.04 g/kg of soil)			12.53 ± 0.95

* According to Tukey’s test results, the data show significant statistical differences (*p* = 0.05).

**Table 2:** Polyaromatic hydrocarbons loss on an example of fluoranthene, fluorene, and pyrene during bioremediation of soil contaminated with creosote oil by PUF-immobilized mycelium of *B. adusta* DSM 3375.

PAH		Absorption Maximum [nm]	Bioremediation Time [Weeks]		
	Sample		5	10	15
			Polyaromatic Hydrocarbons Loss [%] *		
Fluoranthene	PUF with mycelium	302	65.628 ± 0.39	73.185 ± 2.10	79.309 ± 1.24
	PUF without mycelium		2.730 ± 0.13	4.508 ± 0.18	7.807 ± 0.63
Fluorene	PUF with mycelium	299	58.268 ± 1.69	66.564 ± 2.18	71.738 ± 2.51
	PUF without mycelium		3.175 ± 0.17	5.158 ± 0.23	6.685 ± 0.40
Pyrene	PUF with mycelium	297	58.297 ± 1.80	65.351 ± 1.92	72.745 ± 2.36
	PUF without mycelium		2.968 ± 0.97	4.581 ± 0.65	8.589 ± 1.53

* According to Tukey’s test, results obtained for all samples during bioremediation are statistically significant (*p* = 0.05).

**Table 3:** Physical and chemical properties of the soil used in the experiments.

Texture [%]		Ref.
Sand	51	[49]
Clay	15	
Silt	34	
**Moisture [%]**		**Ref.**
25		[50]
**Main Elements [%]**		**Ref.**
Fe	0.039	[51,52]
P	0.032	
N	0.074	
K	0.055	
Ca	0.050	
Mg	0.040	
**pH**		**Ref.**
6.15		[53]

## Data Availability

Not applicable.
